# Comment on “Examining the association between sleep apnea and total hippocampal volumes in cognitive impairment”

**DOI:** 10.1002/alz.70601

**Published:** 2025-08-13

**Authors:** Fangbo Lin, Le Xiao

**Affiliations:** ^1^ Rehabilitation Medicine Department The Affiliated Changsha Hospital of Xiangya School of Medicine Central South University (The First Hospital of Changsha) Changsha China; ^2^ Neurology Department Fujian Medical University Union Hospital Fuzhou China

1

Dear Editor,

We commend Settimi et al. for addressing an increasingly pertinent question—whether obstructive sleep apnea (OSA) accelerates hippocampal atrophy in patients with cognitive impairment of neurodegenerative, vascular, or mixed (NVM) origin.^1^ The study's key strengths deserve recognition.

First, there was a well‐characterized cohort. All participants underwent in‐laboratory polysomnography and three‐dimensional volumetric magnetic resonance imaging (MRI), providing objective, high‐quality exposure and outcome measures.

Second, there was robust image processing. Hippocampal volumes were obtained with an automated, bias‐corrected FreeSurfer pipeline that is widely validated in multicenter studies.

Third, the study had clinical salience. By focusing on a modifiable comorbidity, the authors highlight a pathway that could, in principle, be targeted to slow neurodegeneration.

We believe that certain aspects of the interpretation and discussion merit further exploration to provide a more comprehensive understanding of the findings.

First, only 166 of 279 screened patients were analyzed, largely because of missing MRI or clinical data. Reporting the baseline characteristics of excluded individuals—or applying inverse probability weighting—would help readers judge whether the findings extend to typical memory clinic populations.

Second, men accounted for 57% of the cognitively impaired group and 64% of the OSA group, whereas dementia overall is more prevalent in women.^2^, ^3^ A sex‐stratified analysis (or a sex × OSA interaction term) would clarify whether the reported associations hold across sexes.

Third, stroke history and vascular risk factors varied among diagnostic subgroups, yet quantitative markers of small vessel disease (e.g., white matter hyperintensity volume) were not modeled. Incorporating these metrics in future work will help disentangle OSA‐specific effects from concomitant cerebrovascular injury.

Fourth, hippocampal volumes were derived from three scanners of differing field strengths (1.5 and 3 T). Treating the scanner as a random factor or reporting phantom‐based coefficients of variation would increase confidence that observed differences are biological rather than instrumental.

Last, the composite threshold (apnea–hypopnea index ≥ 15 events·h^−1^, or 5–14 with nadir SpO_2_ ≤ 88%) may misclassify older adults whose dominant burden is sleep fragmentation with milder desaturations. Presenting continuous hypoxic burden measures (e.g., T90%, mean SpO_2_) and exploring threshold–response relationships will clarify the level of exposure at which hippocampal vulnerability becomes clinically meaningful.

Future directions include longitudinal designs that track hippocampal change before and after OSA treatment, integration of quantitative cerebrovascular imaging, and exploration of sex‐specific mechanisms. Such studies could ultimately define the threshold—and the therapeutic window—at which treating OSA yields meaningful neuroprotection.

We hope this balanced perspective, acknowledging both the study's innovations and its limitations, will help readers appreciate the work's contribution while charting a clear agenda for future research.

## CONFLICT OF INTEREST STATEMENT

The authors declare no conflicts of interest. Author disclosures are available in the supporting information.

## Supporting information



Supporting Information
